# Structure Shapes Dynamics and Directionality in Diverse Brain Networks: Mathematical Principles and Empirical Confirmation in Three Species

**DOI:** 10.1038/srep46606

**Published:** 2017-04-20

**Authors:** Joon-Young Moon, Junhyeok Kim, Tae-Wook Ko, Minkyung Kim, Yasser Iturria-Medina, Jee-Hyun Choi, Joseph Lee, George A. Mashour, UnCheol Lee

**Affiliations:** 1 Center for Consciousness Science and Department of Anesthesiology, University of Michigan Medical School, USA; 2 Department of Physics, Pohang University of Science and Technology, Pohang, Republic of Korea; 3 National Institute for Mathematical Sciences, Daejeon, Republic of Korea; 4 Montreal Neurological Institute, McGill University, Canada; 5 Korea Institute of Science and Technology, Seoul, Republic of Korea

## Abstract

Identifying how spatially distributed information becomes integrated in the brain is essential to understanding higher cognitive functions. Previous computational and empirical studies suggest a significant influence of brain network structure on brain network function. However, there have been few analytical approaches to explain the role of network structure in shaping regional activities and directionality patterns. In this study, analytical methods are applied to a coupled oscillator model implemented in inhomogeneous networks. We first derive a mathematical principle that explains the emergence of directionality from the underlying brain network structure. We then apply the analytical methods to the anatomical brain networks of human, macaque, and mouse, successfully predicting simulation and empirical electroencephalographic data. The results demonstrate that the global directionality patterns in resting state brain networks can be predicted solely by their unique network structures. This study forms a foundation for a more comprehensive understanding of how neural information is directed and integrated in complex brain networks.

Recent empirical observations suggest that brain network structure modulates the computation, dynamics, and causal interactions of regional neurons in distinctive ways^1^,^2^,^3^,^4^,^5^,^6^,^7^,^8^,^9^,^10^. Neural activity in hub regions with relatively high connectivity is slower and has more stable dynamics, whereas peripheral regions with less connectivity show faster activity and unstable dynamics^1^,^2^,^3^. The regional difference of neural activities in the time domain is critical to the organization of brain functions. Characteristic hub-periphery neural activities are observed across different species^1^,^4^, giving rise to important questions. For example, does the temporal organization of neural activities across cortical areas arise from the structural organization alone, despite the significant effect of intrinsic local neural dynamics? Furthermore, is there a mathematical principle to explain the role of network structure on temporal organization across cortical areas? In this study, we address these questions by identifying a mathematical relationship between network elements such as node degree, time delay, local synchronization, and(lead/lag) local dynamics.

In a previous study, we proposed a mathematical relationship between network topology, local dynamics, and directionality^11^. A general coupled oscillator model(Kuramoto-type) and *mean field approximation*(MFA) were used for the analysis^12^. Analytical results successfully explained the typical spatial patterns of phase lead/lag neural activities observed in resting and anesthetized states of the human brain^12^. However, the mathematical analysis provided only an inequality relationship—i.e., *if* a node has a larger degree *then* the node will have lagged activity. What is needed to establish a principled structure-function relationship is a method by which to determine the exact phases of coupled oscillators from network elements alone. In this study, we improve upon the fundamental limitation inherent in the MFA method, which averages out the local differences in the connectivity for each node. Motivated by Shima *et al*. and Restrepo *et al*.^13^,^14^, we apply the *local order parameter method*(LOP), which takes into account the details of the local connections for each oscillator^13^,^14^,^15^. Taking into account the local connectivity structure enables us to calculate the precise phases of neural activities and the directionality within a complex, heterogeneous network. The method is then utilized to derive the global directionality patterns in anatomical brain networks from human, macaque, and mouse. The estimated directionality, that is, the lead/lag relationship among regional dynamics, explains how the typical temporal hierarchies of regional brain activities emerge in the brain networks of three species.

## Results

**Figure 1:**
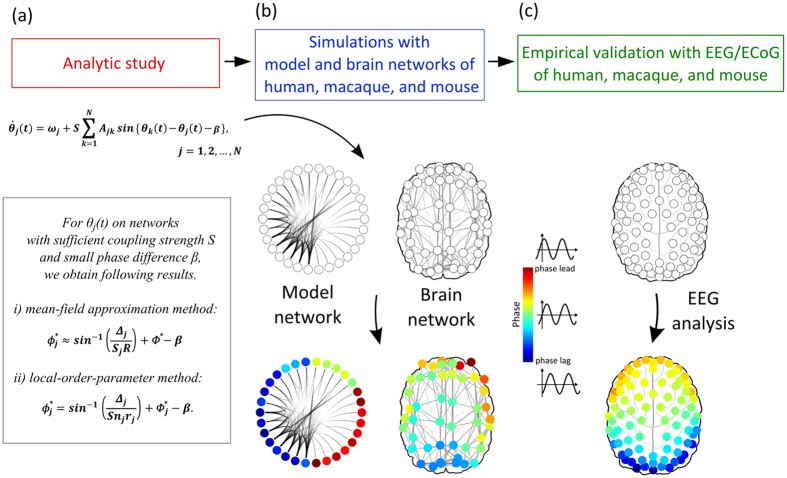
Schematic of analysis. To find a mathematical relationship between network structure and directionality, we performed an analytical study with a coupled oscillator model, numerical simulation, and empirical confirmation.(**a**) In the analytical study, we developed a novel method that calculates the behaviors of oscillators in an inhomogeneous network, in order to derive a mathematical relation between network structure and directionality.(**b**) In the simulation study, we compared the analytical results with the simulation results of homogeneous/inhomogeneous model networks and diverse brain networks.(**c**) In the empirical data analysis, we compared the directionality from the analytical calculation and the directionality in the electroencephalography/electrocorticography(EEG/ECoG) networks of human, macaque, and mouse.

In brief, we first identify mathematical relationships by applying the Kuramoto model to general complex networks. We then use models based on real data from brain networks to compare with experimental data. For the brain models, diffusion tensor imaging(DTI) was used to reconstruct anatomical brain networks of the three species and the Kuramoto model was used to represent neural activities at each node. DTI is a magnetic resonance imaging-based technique, which makes it possible to estimate the density of white matter fibers across brain regions. The density of white matter fibers was utilized to construct macro-scale anatomical brain networks. In these brain networks, a node corresponds to a brain region and an edge corresponds to the density of the structural fiber connection^16^,^17^. The theoretically estimated directionality for each brain network was compared with simulation results and also with EEG data analysis. Because of the simplicity of the model, the brain network simulation and the EEG network analyses are focused on a specific frequency band for each species, in which the largest spectral power of EEG appears: alpha band(7–12 Hz) for primate brains, and theta band(in this case, 6–10 Hz) for the mouse. These consistently-observed frequencies over all EEG channels enable us to extract reliable phases and directionality from EEG. The overall analytical scheme of this study is summarized in Fig. 1.

### Identification of a mathematical relationship between phases of oscillators and network structure

The first aim of this study is to establish a rigorous relationship between underlying network structure and the dynamics of nodes as reflected by coupled oscillators. We start by constructing a general coupled oscillator model on a network. The oscillator at each node of the network represents activities of neural masses in each region of the brain. We adopt a general limit cycle oscillator to represent such activities, motivated by previous work in the literature^5^,^18^,^19^,^20^,^21^,^22^,^23^,^24^. We choose a phase-only oscillator model to focus on phase lead/lag relationships between the oscillators. We use a Kuramoto-type model^23^,^24^,^25^,^26^ as follows:


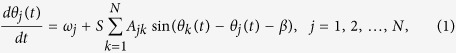


where *θ*_*j*_*(t)* is the phase of oscillator *j* at time *t*, and *ω*_*j*_ is the initial frequency of oscillator *j. S* is the coupling strength between the oscillators, *N* is the total number of oscillators, and *A*_*jk*_ is the coupling from oscillator *k* to *j*, incorporating the structure of the underlying network. *β* is the phase offset, corresponding to the time delay between nodes^27^. Equation(1) describes the time evolution of the phase of oscillator *j*. In general, at a sufficient coupling strength, a system of near-identical coupled limit-cycle oscillators can be approximated by this general phase model^23^. Equation(1) is the *canonical model* for coupled oscillators as the first-order approximation of more complex coupled oscillatory systems(see Methods for the details)^23^,^24^.

Our goal is to obtain the phase of the oscillators *θ*_*j*_ exactly. By utilizing the MFA method and self-consistency argument exploited in our previous work^11^,^12^, we approximate equation(1) to:


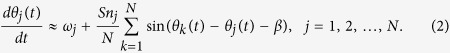


The coupling between nodes *SA*_*jk*_ is replaced with the average coupling strength to the oscillator *j*, defined as the overall coupling strength *S* times *n*_*j*_ where *n*_*j*_ is the sum of the coupling to the oscillator 
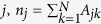
. We then introduce following quantities: 
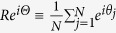
, and Δ_*j*_ *≡* *ω*_*j*_ − *Ω* where *Ω* is the frequency of the population oscillation after the system approaches to the stationary state*. R* is the *global order parameter* showing the degree of synchrony: this parameter ranges from 0 to 1, with 0 meaning uniform incoherence and 1 perfect synchrony. Δ_*j*_ gives the difference between the natural frequency of the oscillator *j* and the frequency after the coupling. We also transform from the original reference frame to a rotating reference frame to make the analysis simpler. If we choose *Ω* as the angular frequency of this rotating frame, we can define *relative phase ϕ*_*j*_ = *θ*_*j*_ *−* *Ωt* representing the phase of the oscillators relative to the average oscillation of the system. Finally, the node *j* with sufficiently large coupling *Sn*_*j*_ > Δ_*j*_ /R asymptotically approach a stable solution *ϕ*_*j*_^∗^ obtained from the following equation:


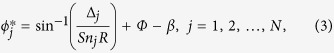


where *Φ* = *Θ* − *Ωt*, which can be set to 0 in our choice of Ω as the angular frequency of the rotating frame(see Supplementary Text for the derivations). We note that for all experimental analysis, phase is measured as the *relative phase* defined as above. Since the inverse sine function in equation(3), sin^−1^(∙), is monotonically increasing from −1 to +1 in the possible domain from −π to π, the value of phase *ϕ*_*j*_ gets larger as the input of the function {Δ_*j*_*/(Sn*_*j*_ *R*)} gets larger. Therefore, a node with a larger *degree n*_*j*_ will have a smaller phase *ϕ*_*j*_.

### Local order parameter(LOP) method improves the limitation of mean-field approximation(MFA) in a heterogeneous network

We apply the *LOP method*, motivated by Shima *et al*. and Restrepo *et al*.^13^,^14^, to derive the phases of coupled oscillators precisely. Similar methods have been applied to other physical systems^13^ and/or to other Kuramoto-type models^14^. Shima *et al*. had used a similar method for a reaction-diffusion system, and Restrepo *et al*. had used the method for a Kuramoto model without time-delay to analyze phase transitions. We first define local order parameter from 
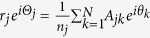
, where *n*_*j*_ is the sum of the couplings to oscillator 
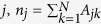
. Local order parameter *r*_*j*_ measures the phase synchrony of the oscillators connected to *j*, and *Θ*_*j*_ is the mean phase among these oscillators; *r*_*j*_ is 1 for perfect synchrony and 0 for a completely incoherent state. Using *r*_*j*_, we can rewrite the equation(1) without any approximation as follows:


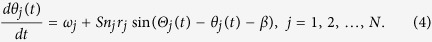


From the equation(4), we derive the exact expression for the phase of oscillator *j*. We again apply the same transformation of the rotating frame. The oscillator *j* with a sufficiently large coupling *Sn*_*j*_*r*_*j*_ > Δ_*j*_ approaches asymptotically a stable solution *ϕ*_j_* of the following equation:


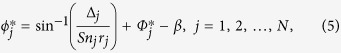


where *Φ*_*j*_^∗^ = *Θ*_*j*_^∗^ − *Ωt*, and in turn 
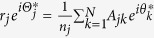
. The equation(5) is the exact solution for the coupled oscillatory model of(4) without MFA(see Supplementary Text for the derivations). For both MFA and LOP methods, the node *j* requires a sufficiently large degree *n*_*j*_(*Sn*_*j*_*R* or *Sn*_*j*_*r*_*j*_ > Δ_*j*_) to result in a phase-locked stable solution. For the node *j* with lower degree, *ϕ*_*j*_ would follow a probability distribution *P(ϕ*_*j*_) instead of having a definitive value at a given time. The probability distribution *P(ϕ*_*j*_) is derived and is described in Supplementary Text. Different from the MFA solution of equation(3), the input of the monotonic increasing function sin^*−1*^(∙) in(5) is {Δ_*j*_*/(Sn*_*j*_ *r*_*j*_)}, that is, the value of phase *ϕ*_*j*_ gets larger as {Δ_*j*_*/(Sn*_*j*_ *r*_*j*_)} gets larger. Therefore, because of the inverse relationship, a node with larger degree *n*_*j*_ and larger local synchrony *r*_*j*_ will have smaller phase *ϕ*_*j*_ and be relatively phase lagged with reference to the average phase of the network. Equation(5) quantitatively shows the determinant role of the local connection on the phase of a node.

### Analytical calculation estimates the simulation results for random and scale free networks

**Figure 2:**
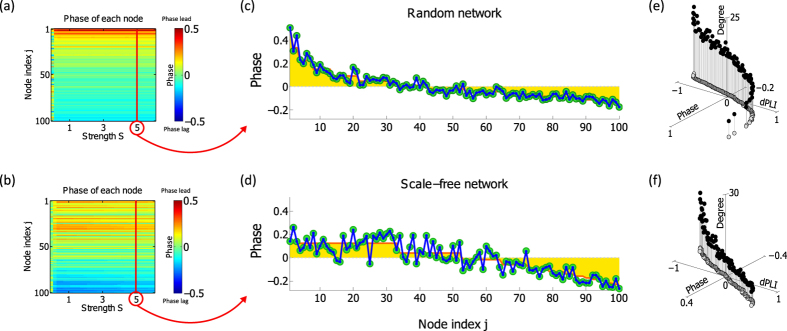
Comparison between the analytical and simulation results for random and scale-free networks. Figure 2(**a**) and(**b**) present the simulation results for random(homogeneous) and scale-free(inhomogeneous) networks. Each figure presents the phase values of 100 nodes as increasing coupling strengths *S*. The nodes are arranged in ascending order of node degree on the y-axis, and the phases are denoted with colors. Red color denotes that the nodes are phase-leading, whereas blue color denotes that the nodes are phase-lagging. The phases are maintained consistently if the coupling strength is large enough(>0.2). The simulation result of the specific coupling strength(S = 5) is presented in(**c**) and(**d**).(**c**) and(**d**) present the analytical results for the mean-field approximation(MFA) and local order parameter(LOP) methods, and the simulation results for the two network models. The nodes are arranged in ascending order of node degree, and the relative phases estimated with different methods are plotted with different colors. Green circle(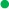) denotes the simulation result, blue line(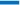) denotes the phase from LOP, and red line(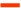) denotes the phase from MFA. A gray dotted line(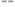) denotes phase = 0. Figure 2(**e**) and(**f**) present the 3d plots of degree *n*_*j*_, phase *ϕ*_*j*_, and *dPLI*_*j*_ of 100 nodes for both networks.

We test how, precisely, the analytical calculations of the MFA and LOP methods estimate the simulations with both random and scale-free model networks. The random network has a homogeneous Poisson degree distribution, whereas the scale free network is inhomogeneous with a power law distribution(which has a hub-periphery structure). First, we conduct a simulation with a network size of 100 nodes, varying the coupling strength *S* from 0 to 6. Figure 2(a) and(b) presents the phases of the 100 oscillators. The 100 nodes in the networks are arranged in the ascending order of node degree. As predicted by the analytical study of the relationship between phase and node degree, the phase lag of higher degree nodes(blue color) and the phase lead of lower degree nodes(red color), is robust as long as the coupling strength is large enough(*S* > 0.2). We chose the specific coupling strength(S = 5) for the direct comparison between the analytical and simulation results for each node. We chose a sufficiently large coupling strength so that the phases of each node would be stable and the maximum correlation coefficient between analytical and simulations results was achieved.

Figure 2(c) and(d) demonstrates the analytically calculated phase *ϕ*_*j*_*(t)* for each node *j* with the MFA(red line) and LOP(blue line) methods, and the numerical simulation(green circle) for the model networks. First, we observe that the hub nodes with higher degrees have lagging phases(negative values) and peripheral nodes with lower degrees have leading phases(positive values). Second, compared to the MFA method, the LOP method estimates the simulation results more precisely. The correlation between the phases of the LOP method and the numerical simulation is almost 1.00(Spearman correlation with p = 0) for both networks, whereas the MFA method has correlations of 0.79 and 0.78, respectively(Spearman correlation with p < 10^−3^). In particular, the LOP method, which takes into account the local connectivity, works well for scale-free network, whereas the MFA method fails.

With the analytically calculated phases, we then estimate the directionality of the networks. As a phase based directionality measure, we use directed Phase Lag Index(dPLI), which measures the asymmetry of phase lead/lag relationship between two time series^28^. When the phases of a time series lead the phases of another time series, the dPLI is 1, and in the opposite case −1. In a network, *dPLI*_*j*_ for a node *j* is defined by the averaged dPLI for all possible pairs of node *j*. By definition, the dPLI_*j*_ and the phase *ϕ*_*j*_ of a node *j* will have a high correlation, but will not be identical(see Supplementary Text for the relationship between dPLI and *ϕ*). Figure 2(e) and(f) present strong correlations between node degree, phase, and dPLI in the random and scale-free networks. The correlations between *ϕ*_*j*_ and *dPLI*_*j*_ are 0.97(Spearman correlation with p = 0) for both networks. The high correlation between three indices implies that the local dynamics(phase) and the directionality(dPLI) in the network are predictable by network connectivity(node degree) alone. These relationships are applied to estimate the directionality of the brain networks.

### Analytical calculation estimates the simulation results for reconstructed brain networks of human, macaque, and mouse

**Figure 3:**
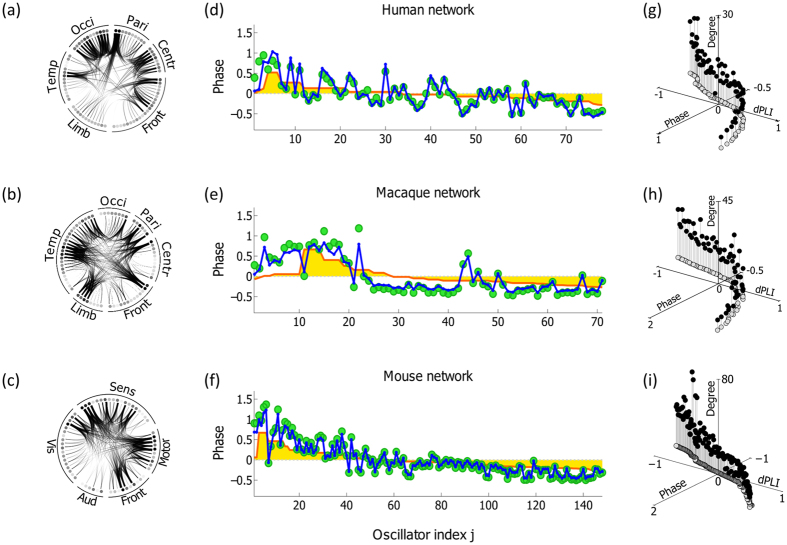
Comparison between the analytical and simulation results for brain networks of three species. The brain network structures of(**a**) human,(**b**) macaque, and(**c**) mouse are depicted using ring plots. The brain networks are separated into several groups: frontal(front), central(centr), parietal(pari), occipital(occi), temporal(temp), limbic(limb), motor(motor), somatosensory(sens), visual(vis), and auditory(aud). The dots and lines in the ring plot denote the nodes and connections in a network. Only the top 30% of node degrees are presented. Figure 3(**d**),(**e**) and(**f**) present the analytical results of mean-field approximation method(red line, 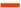) and local order parameter method(blue line, 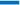), and the simulation result(green circle, 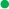) for human, macaque, and mouse. The nodes are arranged in the ascending order of node degree(from periphery to hub) on the x-axis and the relative phases on the y-axis. Figure 3(**g**),(**h**) and(**i**) depict the 3d plots of degree *n*_*j*_, phase *ϕ*_*j*_, and *dPLI*_*j*_ for human, macaque, and mouse.

The brain is a complex network structure with highly inhomogeneous connectivity. The complex connection structure may hinder the precise calculation of the phases. To test whether or not the analytical method works for complex brain networks, we apply the MFA and LOP methods to brain networks reconstructed from DTI tractography of human, macaque, and mouse. The structural networks constructed from DTIs of human and macaque brains were acquired from previous studies, while the structural network of the mouse is constructed from DTI for this study. The human brain network has 78 nodes in the cerebral cortex^16^ and the macaque has 71 nodes only from the left hemisphere in the cortex^29^. The mouse brain network has 150 nodes from the entire brain region including the cortex and subcortical regions(see Methods and Supplementary Text for the DTIs). The brain network structures of human, macaque and mouse are presented with the ring plots in Fig. 3(a,b) and(c). In the ring plot, the top 30% of node degrees are presented, and the dots and lines denote the nodes and connections. Figure 3(d,e) and(f) present the phases calculated with two analytical methods(MFA and LOP) and the phases from the numerical simulation based on the anatomical brain networks. In the analytical calculation and numerical simulation, we focus on a specific frequency band(6~12 Hz), in which the similar peak frequencies are observed in the overall EEG. This focus enabled us to compare the analytical results to the phases of EEG channels in the next section. We note that in the simulation, the results were qualitatively similar for S > 0.2. For the comparison, we again chose the stable phases at S = 5.

The MFA method(orange line) and the LOP method(blue line) show different performances depending on the brain networks. The LOP method estimates the local fluctuations of phases more accurately(Spearman correlation with the numerical simulation result was 0.99 and p = 0, with a mean prediction error of less than 0.1 for all species), whereas the MFA method fails to estimate the local fluctuations. In particular, the MFA method becomes worse for the lower degree nodes(e.g., peripheral nodes with sparse connections in brain networks). The limitation for the lower degree nodes can be mitigated by using the probability distribution function *P(ϕ*_*j*_) derived from equation(5)(See Supplementary Text for the calculation of *P(ϕ*_*j*_)).

Figure 3(g,h) and(i) shows the strong correlations between node degree, phase, and dPLI(Spearman correlations >0.95 and p = 0 among the indices in each network). This result indicates that the local dynamics(node degree) and directionality(dPLI) are predictable from the brain network structure(node degree).

### Analytical calculation estimates the directionality in the EEG-based networks of human, macaque, and mouse

**Figure 4:**
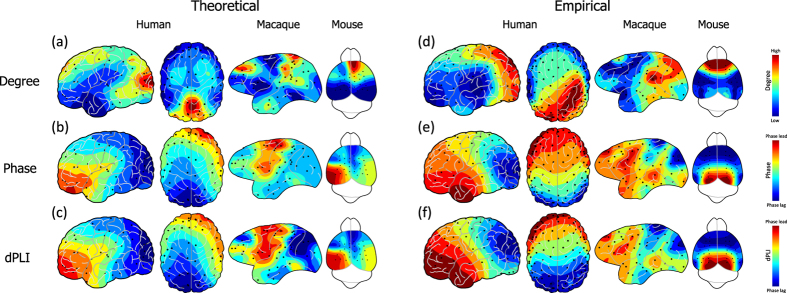
Comparison between the theoretical predictions and the empirical data analysis. Figure 4(**a**),(**b**) and(**c**) are the topographic plots for node degree, phase, and dPLI, estimated with the local order parameter method and the anatomical brain network structures. The node degree distributions of the brain networks have large negative correlations with the distributions of phases and dPLI. The network structure(node degree) of each species can predict the local dynamics(phase) and the directionality(dPLI). Figure(**d**),(**e**) and(**f**) are the topographic plots for node degree, phase and dPLI, which are calculated from EEG/ECoG data. The node degree in the EEG networks has a significant negative correlation with the phase and dPLI in the EEG networks.

We compare the phases, dPLIs, and node degrees from the analytical calculations with DTI and empirical data analysis with EEG/EGoG. For visualization, we map the node degree, phase and dPLI values at each node and extrapolate the values on 2-dimensional brain images(see Supplementary Figure 1). Figure 4(a) demonstrates the node degree distributions of DTI from the three species. Human and macaque brain networks have primary hubs in posterior regions, whereas the mouse has primary hubs in anterior regions. Figure 4(b) and(c) demonstrates prominent(negative) spatial correlations of the phases and dPLIs with the node degrees in Fig. 4(a). In the human network, the asymmetric phase distribution in the frontal-posterior region shapes the directionality from frontal(source, with *dPLI*_*j*_ > 0, red color) to posterior region(target, with *dPLI*_*j*_ < 0, blue color). In the macaque, the overall pattern of directionality is similar, with primary target(dark blue) distributed in the parietal-occipital lobes and the sources(dark red) in the central region. However, the mouse demonstrates an opposite phase distribution and directionality pattern compared with primates.

To verify the analytical results, we analyze the resting-state EEG data acquired from six human volunteers, four macaques, and eight mice. For the six human volunteers, 128-channel EEGs are recorded with eyes closed. After removing noisy channels, 106 EEG channels are selected across the subjects for the analysis. For the macaques, 128-channel ECoG was recorded from the left hemisphere with eyes closed. Since the four macaques have different channel locations, we divide the hemisphere into 48 regions using a parcellation scheme introduced by Lewis and van Essen for monkey^30^, and calculate the average value for each region. These 48 regional values are compared across macaques(see Supplementary Figure 2 for details). For the mice, 38-channel EEG was recorded during the quiet waking state; 32 channels are selected for the analysis after removing noisy channels. Graph-theoretical network analysis was applied to construct functional brain networks from the EEG/ECoG data. *Phase lag index*(PLI)^31^, which is a measure of phase locking between two signals, is calculated between all pairs of EEG/ECoG channels and used for weighted edges. The node degrees in the EEG/ECoG networks are presented in Fig. 4(d).

**Figure 5:**
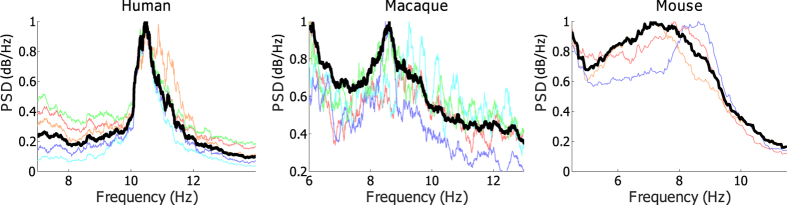
Power spectrograms of human, macaque, and mouse. Figure 5 shows representative power spectral densities(PSD) of the exemplary subjects from human(**a**), macaque(**b**), and mouse(**c**). The peak frequency in the PSD is used to determine specific frequency bands for each individual to extract reliable phases from the EEG signals. The bold black line(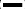) represents the mean PSD over all channels, and the different colors represent the average regional PSDs. The frequency bands can be selected around 10.5 Hz, 8.5 Hz, and 7.5 Hz for these individuals.

Through examining the broad frequency range(0.5–55 Hz), we find across the three species that the frequency band including the highest peak of the power spectrum yields robust lead/lag phase relationship in the EEG networks. The highest peaks for human, macaque and mouse are observed in the frequency range from theta to alpha band(6–12 Hz). To calculate reliable and robust phase and dPLI from EEG/ECoG data, we chose the frequency band around the highest peak(±2 Hz around the peak) for each subject. The peak frequencies of the three species are presented in Fig. 5.

Figure 4(e) and(f) shows the phase *ϕ*_*j*_ and *dPLI*_*j*_ calculated with the selected frequency bands. As predicted, the spatial distributions of node degrees and dPLIs in Fig. 4(d) and(f) have significant correlations for the three species(Spearman correlations are −0.70, −0.53, and −0.45 for human, macaque, and mouse, respectively, with p < 0.001). These results imply that we can estimate the directionality(dPLIs) with the node degree distribution in neurophysiologically-derived networks. The empirical results are also interpretable based on the mathematical relationship we found in equation(5). However, the inconsistent prediction performance across the species(for instance, the human EEG exhibited a higher correlation compared to the mouse EEG) may be due to different qualities of EEG/ECoG data: the human subjects were under well-controlled conditions with dense EEG channels, which covers the whole scalp, whereas the mice were in a quiet waking state with sparse EEG channels. It is also notable that the DTI and EEG were from different subject groups. This limitation may cause a large individual variability in the comparison between the theoretical estimations((b) and(c)) and the empirical data analysis results((d) and(f))(0.89: the largest coefficient between LOP and EEG for the dPLI of human, and 0.28: the smallest coefficient between LOP and EEG for the phase of macaques, and the other results are presented in Supplementary Figure 3). All individual topography from experimental data analysis is presented in Supplementary Figures 4–7.

In addition, we performed simulations with heterogeneous time delays between nodes. The time delays between each node were given proportional to their Euclidean distances. The results yield similar or higher correlation coefficients between model simulations and empirical data analysis results, suggesting the possibility of a more realistic model with additional variables(0.82, 0.45, 0.63 for dPLI of human, macaque, and mouse, respectively, and 0.76, 0.49, 0.63 for the phase of each species). The topographic figures and the correlation coefficients are shown in Supplementary Figure 8.

## Discussion

Human, monkey, and murine species have anatomical and functional brain networks with a characteristic hub and peripheral node organization that varies based on species^16^,^17^,^29^,^30^,^32^,^33^,^34^,^35^,^36^,^37^,^38^,^39^,^40^. Across various species, Van den Huevel *et al*. observed conserved wiring principles such as community structure and long-range connections^17^. Community structure reflects functional specialization, whereas long-range connection supports short communication paths. The competition between efficient topological integration and economic wiring may shape the possible topology of the brain network^41^. Dynamically, the hub-periphery structure is reflected in the functional network by complex interactions between local neural masses^4^,^17^,^42^. Several studies have examined the relationship between structural and functional networks through a principled approach^10^,^24^,^43^. For instance, Honey *et al*. and Tewarie *et al*. studied how structural networks can influence functional networks^3^,^5^,^8^. Marinazzo *et al*., Stam *et al*., and Rabinovich *et al*. studied information flow and directionality in brain network models^10^,^28^,^43^. Several studies used oscillatory models to simulate brain activities^18^,^19^,^20^,^21^,^22^. Building upon our previous investigation^11^, in this study we derived and applied mathematical analysis to the relationship between node degree, time delay, local synchronization, and phase of oscillators in a network, analytically explaining the effect of each network element on local node dynamics. The comparison between our theoretical prediction and the empirical data demonstrated clearly that the brain networks of the three species(human, macaque, and mouse) shape their characteristic phase distributions and directionality patterns in a way predicted by their individual topology.

In the analytical study, we identified how network structure modulates initially-identical oscillators in different ways. After modulation, a higher degree node has a smaller phase; in contrast, a lower degree node has a larger phase. In terms of directionality, the higher degree node(hub) acts like a target, whereas the lower degree node(periphery) acts like a source. We also improved upon the limitations of the MFA method, which assumes all nodes are connected to one another and thus allows only a coarse-grained approximation. The LOP method can capture the local fluctuation of phase due to the heterogeneous local connectivity patterns. The LOP method also makes it possible to derive the exact phases of the oscillators and their corresponding directionality pattern in a heterogeneous network, whereas the MFA method cannot. These results can be considered as the first-order approximations of more realistic neural-mass models, providing insights into more complex network dynamics^11^,^23^,^24^,^44^,^45^. As a next step, it will be beneficial to expand the methods to amplitude-phase models and excitatory-inhibitory neuronal models(e.g., Stuart-Landau and Wilson-Cowan models). In particular, the spatial interaction patterns of amplitude and phase from the models would reveal more realistic network dynamics in the brain. The directionality of amplitude and phase dynamics in a brain network could be different and potentially in the opposite direction.

From the empirical data analyses of human, macaque, and mouse, we characterized the phase and directionality patterns of each species. Human and macaque had a similar phase distribution pattern: larger and smaller phases in frontal and posterior regions, respectively; in contrast, the mouse had the opposite pattern of smaller and larger phases in frontal and posterior regions, respectively. Accordingly, human and macaque showed large-scale frontal-to-posterior directionality, with the inverse directionality for mice. Despite varying results, these empirical observations of phases and directionality patterns across species could be successfully predicted by the anatomical brain network structure and the mathematical relationship we identified. In the human brain, the prominent high degree hub structure in the posterior region is associated with relatively lagged phases and is a directional target. By contrast, the relatively lower-degree nodes in the frontal region are associated with leading phases and a directional source. Because of the similar anatomical hub/periphery structures, macaque has a similar pattern compared with human. However, the opposite hub/periphery structure of the mouse produces the opposite phase and directionality patterns from human and macaque. Notably, despite the intrinsic activities of local regions, the analytical results estimated with the anatomical brain network structures successfully explained the phases and directionality patterns of EEG/ECoG networks. Collectively, the analytical, simulation and empirical data analysis explained how the brain network of each species integrates spatially distributed information with a typical temporal hierarchy. In terms of practical application, this approach is relevant to various clinical cases for estimating the potential change of the temporal hierarchy of regional brain activities(the lead/lag relationship of phases) at the time of brain injury or intervention, providing a recovery strategy in a principled way(e.g., through the use of stimulation).

There are a number of limitations to this study. First, the simple coupled Kuramoto model enabled an analytical study, but the simplicity limits our results to large-scale temporal and spatial behavior, i.e., relatively long-term and macroscopic network dynamics. Since the local dynamics may be dominant in micro- or mesoscale networks, it is unclear to what extent our analytical result is applicable to other scales. Second, our analysis primarily focused on the dynamics of a specific frequency band(6–12 Hz), which includes clear peaks in the power spectrum from the EEG data. With the frequency band of 6–12 Hz, the theoretical prediction of the relationship between global network topology and node dynamics correlated well with the empirical data. It also suggests that the dominant oscillations may be associated with global neural activities across the entire brain network structure. Third, the phase lead/lag relationship of coupled neurons may not directly reflect their directionality. For instance, anticipated synchronization provides a counter example, showing that under a negative feedback loop condition, the phase of the receiver neuron leads the phase of the sender neuron in time. However, in our previous study, we showed with model data that the mean phase lead/lag relationship between two nodes correlates with causal relationships as reflected by granger causality and symbolic transfer entropy^11^,^46^,^47^. In this study, we chose dPLI, a measure of asymmetry of phase lead/lag relationship^28^ rather than a more sophisticated causality measure. Because of its simple form, we could derive a mathematical relationship and directly compare the analytical results to empirical data. Fourth, the anatomical networks we used for theoretical predictions were not from the same individuals in whom we recorded the EEG/ECoG. The mismatch between theoretical prediction and empirical data analysis could have arisen, in part, from this difference. The analytical estimation would be more accurate with more subjects or simultaneous recordings of both anatomical network and EEG for the same individual. Lastly, considering that EEG records superficial brain activities, the MFA(a coarse grain method) could outperform the LOP(a more sophisticated method) with these data sets.

In conclusion, we identified a mathematical relationship between network elements such as node degree, time delay, local synchronization, and(lead/lag) local dynamics. The application of the mathematical relationship to the brain networks of human, macaque, and mouse, explained how the brains of three species shape the distinctive directionality patterns in their network structures. The analytical method and the mathematical relationship are applicable to other networks across different disciplines, estimating properties of function from structure.

## Methods

### Kuramoto Model

To identify the relationship between network topology and the dynamics of nodes in inhomogeneous networks, we used a general coupled oscillator model. A general form of coupled oscillatory activities in a network is presented as a phase variable:


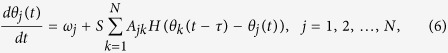


where *τ* is the finite transmission delay between oscillators, emulating the delay of signal propagation between neural mass populations. Equation(6) gives a time evolution of the phase of oscillator *j, θ*_*j*_. In general, for small coupling strength *S*, a complex oscillatory system can be reduced to a simple phase model. The sine function was used for the coupling function, *H*(∙) = sin(∙), converting the equation(6) into the coupled Kuramoto oscillator model. We also used the short time delay approximation by Izhikevich, which states that small time delays can be approximated by phase offset term^27^, thus translating the time delay *τ* into the corresponding phase offset *β*. These considerations guided the derivation of our model equation(1).

### Simulation Parameters

In our simulation, the natural frequencies of the oscillators are given as *f* = 10 Hz, making *ω*_*j*_ = *f*∙2π rad/s. Three types of delays were given in simulations:(a) an identical phase offset *β* of small value between coupled nodes,(b) an identical time delay of 4ms and 10 ms between coupled nodes, or(c) diverse time delays proportional to the physical distances between nodes, which are between 4 to 10 m/s(for brain network simulation only). For all types of delay the results were qualitatively similar and the quantitative differences were small. Increasing the coupling strength from 0 to 6, no qualitative difference was observed. For all simulations, we added Gaussian white noise *ξ*_*j*_*(t)*(mean and standard deviation of 1) in order to test the robustness of simulation result against random fluctuations. Gaussian noise did not change the simulation results qualitatively. For a given parameter set, the measurement was averaged over at least 1,000 runs of the simulation.

### Phase Lag Index and Directed Phase Lag Index

We calculated *directed Phase Lag Index*(dPLI) between nodes *i* and *j* to determine the phase-lead/phase-lag relationship between channels^28^. dPLI between two nodes *i* and *j* can be defined as:


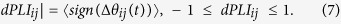


Here, Δ*θ*_*ij*_*(t)* is the instantaneous phase difference between two nodes *i* and *j*: Δ*θ*_*ij*_*(t) *=* θ*_*i*_(*t)- θ*_*j*_*(t)*. The sign() function yields 1 if Δ*θ*_*ij*_*(t)* > 0, −1 if Δ*θ*_*ij*_*(t)* < 0, and 0 if Δ*θ*_*ij*_(*t*) = 0. The mean is taken over all *t* = 1, 2, 3, …, *n*. Therefore, on average, if node *i* leads node *j*, 0 < *dPLI*_*ij*_ ≤ 1; for the inverse, −1 ≤ *dPLI*_*ij*_* *<* *0; and if there is no phase-lead/phase-lag relationship, *dPLI*_*ij*_ = 0. *dPLI*_*i*_ for a node *i* was defined as the average of *dPLI*_*ij*_ for all other nodes *j*.

The absolute value of *dPLI*, called *Phase Lag Index(PLI*), measures the phase locking of two signals^31^. If the instantaneous phase of one signal is consistently ahead or behind of the other signal, the phases are considered locked, and PLI_*ij*_ ≈ 1. However, if the signals randomly alternate between a phase-lead and a phase-lag relationship, there is no phase locking, and PLI_*ij*_ ≈ 0.

### Complex model networks

The coupled oscillatory model was applied to both random and scale-free networks with various sizes of nodes(100, 1000, and 2000). For the random network, the Gilbert algorithm was used with the parameter of *G(N,(1* + *ε)log(N)/N)*, where *N* is the number of nodes, and *ε* is an arbitrary small number. The Catanzaro *et al*.’s algorithm was used to generate a randomly connected network with scale-free node degree distribution given a priori^48^. The scale-free networks with varying slopes of the degree distribution from −2 to −3 were used for the simulations. Our analysis predicted correctly the phase of each oscillator regardless of the size and type of network.

### Anatomical brain network of human, macaque, and mouse

The human brain network was constructed from diffusion tensor imaging(DTI) of 80 young adults^16^. The network consisted of 78 parcels of the cerebral cortex. The macaque brain network consisted of 71 parcels of the left hemisphere in cerebral cortex^29^. The mouse brain network was constructed for this study with DTI tractography of eight adult mice. The DTI of the adult mouse are available at the Johns Hopkins Medical Institute, Laboratory of Brain Anatomical MRI^35^. For the details of the mouse brain network construction, see Supplementary Text.

### Experimental data from human, macaque, and mouse

The Human EEG recording(6 subjects age between 25 to 27, three males) was conducted at the University of Michigan Medical School and was approved by the Institutional Board Review(HUM00061087); written consent was obtained from all participants after a discussion of risks and benefits. After IRB approval and written informed consent, 128-channel EEG with sampling rates 500 Hz was recorded continuously for one session each from six healthy subjects in the resting state with eyes closed. Sensor net from Electrical Geodesics Inc. was used for the EEG acquisition. 180-second artifact-free epochs were analyzed for each session. All channels were referenced to the vertex with electrical impedance reduced to below 50 kΩ(as per manufacturer recommendation) prior to data collection. After the recording, EEG signals were high pass filtered at 0.1 Hz, and re-referenced to an average reference. Subsequently, because of different species and different recording environments we did not use common criteria to remove artifacts in EEG data, instead, we visually inspected and excluded the epochs containing artifacts such as eye blink and muscle artifacts. A total of 106 EEG channels were used for the analysis, covering prefrontal, frontal, central, temporal, parietal, and occipital areas.

For macaques(three are M. Fuscata, and one is M. Mulatta), the data were acquired from Project Tycho(http://neurotycho.org/)^49^. 128-channel ECoG of the left hemisphere was recorded continuously for 11 sessions in total from four healthy subjects in resting state. The sampling rate was 1 kHz and 540-second artifact-free epochs were analyzed for each session. A ground electrode and a reference electrode were located in the epidural space and in the subdural space between the ECOG array and dura matter, respectively. Since the four monkeys had different spatial locations of ECoG channels from one another, we divided the hemisphere into 48 regions using the parcellation scheme of Lewis and van Essen^24^ in order to make the ECoG channels comparable across the monkeys. The regional averages over the ECoG channels were considered to represent the newly divided 48 regions. Noisy channels were excluded. All experimental and surgical procedures were performed under the approval of RIKEN ethics committee and the recommendations of the Weatherall report(see Nagasaka *et al*.^50^, for details).

For mice, 38-channel EEG with a sampling rate of 500 Hz was recorded continuously for 16 sessions in total from 8 healthy subjects during the quiet waking state. 30-second artifact-free epochs were analyzed for each session. The reference and ground electrodes were fixed onto the skull above the right cerebellum and the right olfactory bulb, respectively. EEG channels contaminated by artifact were excluded by visual inspection; the remaining 32 EEG channels that were common across subjects after removing noisy EEG channels were used for analysis. All experimental and surgical procedures were conducted in accordance with the guidelines for the Institutional Animal Care and Use Committee of the Korea Institute of Science and Technology(KIST), following Act 1992 of the Korea Lab Animal Care Regulations and associated guidelines(see Choi *et al*. and Lee *et al*. for more details^51^,^52^).

### Network analysis of EEG/ECoG

The node degree and dPLI for each node were calculated in EEG/ECoG networks constructed from each species. First, the EEG/ECoG was segmented into 5-second epochs to establish a pseudo-stationary state. The averaged values for node degree, amplitude, and dPLI over all the segmented epochs represented the individual(the number of segments are 36 for human, 108 for macaque, and 6 for mouse). For each segmented epoch, the band pass filter was applied for six frequency bands. Band-pass filtering with the fifth-order Butterworth filter was applied to EEG forward and backward, correcting the potential phase shifting after band-pass filtering(“butterworth.m”, and “filtfilt.m” in Matlab; MathWorks, Natick, MA). For phase calculation of signals from channels, Hilbert transform was performed to extract phase information at each time point for each frequency band, and then relative phase for each channel was calculated. The PLI was calculated for all pairs of EEG channels and the adjacency matrix was constructed using the PLI values as the weighted edges. The specific threshold was chosen by searching for the best-fit to the simulation and testing the robustness over different thresholds. Node degree for each channel was computed from the binary network, which counts the number of links connected to a node. dPLI for a channel was computed with averaged dPLI between the given channel and all other EEG/ECoG channels. Consequently, for each EEG/ECoG epoch, we were able to obtain the node degrees and dPLIs for all EEG/ECoG channels. The Spearman correlation coefficient was used for evaluating the correlations between node degree and dPLI of each channel(“corr.m” in Matlab).

## Additional Information

**How to cite this article**: Moon, J.-Y. *et al*. Structure Shapes Dynamics and Directionality in Diverse Brain Networks: Mathematical Principles and Empirical Confirmation in Three Species. *Sci. Rep.* **7**, 46606; doi: 10.1038/srep46606(2017).

**Publisher's note:** Springer Nature remains neutral with regard to jurisdictional claims in published maps and institutional affiliations.

## Supplementary Material

Supplementary Text and Figures
