# Supplementary material for: Structure Shapes Dynamics and Directionality in Diverse Brain Networks: Mathematical Principles and Empirical Confirmation in Three Species
Source: Sci Rep. 2017 Apr 20;7:46606. doi: 10.1038/srep46606 (PMC5397857; doi:10.1038/srep46606)
Supplement: Supplementary Text and Figures [file srep46606-s1.pdf]

Structure Shapes Dynamics and Directionality in Diverse Brain Networks: Mathematical Principles and Empirical Confirmation in Three Species

Supplementary Text

Joon-Young Moon[1], Junhyeok Kim[2], Tae-Wook Ko[3], Minkyung Kim[2], Yasser Iturria Media[4], Jee-Hyun Choi[5], Joseph Lee[1], George A. Mashour[1], and UnCheol Lee[1]

[1]Center for Consciousness Science and Department of Anesthesiology, University of Michigan Medical School, USA
[2]Department of Physics, Pohang University of Science and Technology, Pohang, Republic of Korea
[3]National Institute for Mathematical Sciences, Daejeon, Republic of Korea
[4]Montreal Neurological Institute, McGill University, Canada
[5]Korea Institute of Science and Technology, Seoul, Republic of Korea

Contents

1 Derivation of phases for the Kuramoto model in complex networks **2**
Kuramoto model as a general model for a system of coupled oscillators in networks 2
Mean-field approximation method applied to the model 4
Local order parameter method applied to the model 7
Relationship between directed Phase Lag Index and phase 11

2 Experimental analysis **12**
Construction of the mouse structural network 12

3 Supplementary Figures Information **14**

Here we provide details regarding the analysis summarized in the main manuscript. In section 1, we derive the results of the mathematical analysis performed for the coupled oscillator model on complex networks. We begin with the description of the model we use - *the Kuramoto model* - and state that the model is the lowest-order approximation of the more detailed neural mass models and thus can be used to gain insights into how more realistic models behave. We then proceed to the main results: the derivations of the phase of each oscillator in the networks by *mean-field approximation* and *local order parameter* methods. We also provide an argument for the idea that knowing the relative phase of each oscillator is qualitatively equivalent to knowing the *directed phase lag index (dPLI)* with high probability. In section 2, we provide more information about the experimental analysis for brain networks of different species. We begin with the process of constructing the structural brain network of mouse. Lastly, we give descriptions for the Supplementary Figures 1-7.

1 Derivation of phases for the Kuramoto model in complex networks

In this section we describe in detail the mathematical analysis performed for the coupled oscillator model in complex networks. We first start with the statement of the model, then move on to derive the phases for each oscillator with two methods. Lastly, we present an argument for the qualitative equivalence between relative phase and dPLI value for each oscillator.

Kuramoto model as a general model for a system of coupled oscillators in networks

The first aim of this study is to refine the relationship of network topology and the dynamics of oscillators in the network in order to calculate precisely the phase of each oscillator in terms of network measures such as degree. We start by constructing a coupled oscillatory network model with the activity at each node of the network represented as a single phase variable:

$$\dot{\theta}_j(t) = \omega_j + S\sum_{k=1}^{N} A_{jk} H(\theta_k(t-\tau) - \theta_j(t)), \qquad j = 1, 2, ..., N, \tag{S1}$$

where $\dot{\theta}_j(t)$ is the phase of oscillator j at time t, ω_j is the natural frequency of the oscillator j, N is the total number of oscillators, S is the overall coupling strength between oscillators,A_{jk} is the coupling between oscillator k to oscillator j, and τ is the finite transmission delay between different oscillators emulating the delay of signal propagation between two neural mass populations. $H()$ is the coupling function. Equation (S1) is a general form of coupled oscillators with time delays between them, giving the time evolution of the phase of oscillators. At a sufficient coupling strength S, coupled identical oscillatory systems can be reduced to such a phase model in general [1].

Ref. [4] states that for a given coupled oscillatory system with time delays, if the delay between the coupled oscillators is smaller in the order of magnitude compared to the oscillator's oscillatory period, then there will be no explicit time delay: rather, the delay will appear as a simple phase difference term β in the coupling function.

Incorporating the result of ref. [4] and also using sine function as the coupling function, $H() = sin()$, we arrive at the following so-called coupled Kuramoto-type oscillator model:

$$\dot{\theta}_j = \omega_j + S\sum_{k=1}^{N} A_{jk} \sin(\theta_k - \theta_j - \beta), \qquad j = 1, 2, ..., N. \tag{S2}$$

The difference compared to the original Kuramoto model is that now the coupling among oscillators is selectively represented by A_{jk} [2, 3]. This Kuramoto-type oscillator model is the *canonical model* for oscillators, in the sense that it is the first-order approximation to the more general from of equation(S1). In our text we will refer to our Kuramoto-type oscillator model as simply Kuramoto model for the brevity.

We note that, for the purpose of our analysis, the natural frequencies will be larger than zero and the nonzero phase delay β will be small compared to the given natural frequencies ω_j to assume that $\beta \in (0, \pi/2)$. This will be the range of the parameters under our analysis.

We proceed to analytically predict the phases θ_j from equation (S2).

Mean-field approximation method applied to the model

By utilizing the mean-field approximation (MFA) technique and self-consistency argument described in our previous works [5, 6], we can simplify equation (S2). Assuming that the oscillator connections are random, the following approximation is applied [5, 7]:

$$S\sum_{k=1}^{N} A_{jk}H(\theta_k - \theta_j) \approx \frac{Sn_j}{N}\sum_{k=1}^{N} H(\theta_k - \theta_j). \tag{S3}$$

Here, n_j is the sum of coupling to the oscillator j defined as $n_j = \sum_{k=1}^{N} A_{jk}$.

With equation (S3), equation (S2) is approximately equivalent to the following equation:

$$\dot{\theta}_j = \omega_j + \frac{Sn_j}{N}\sum_{k=1}^{N} \sin(\theta_k - \theta_j - \beta), \qquad j = 1, 2, ..., N. \tag{S4}$$

The aim of this subsection is to obtain the solution for θ_j in equation (S4). We first introduce *global order parameter R*:

$$Re^{i\Theta} = \frac{1}{N}\sum_{k=1}^{N} e^{i\theta_k}. \tag{S5}$$

R will have values between 0 and 1. 0 indicates uniform incoherence, and 1 indicates *in-phase synchrony*. In the state of in-phase synchrony, the oscillators of the system will oscillate with same frequency and same phase.

Let Ω denote the frequency of the population oscillation of equation (S5) after the system approaches a stationary state. Also let $\phi_j = \theta_j - \Omega t$ represent the phase of oscillator j relative to the average phase of the system. Equation (S4) can be rewritten using the order parameter as follows:

$$\dot{\phi}_j = \omega_j - \Omega + Sn_jR\sin(\Phi - \phi_j - \beta), \qquad j = 1, 2, ..., N, \tag{S6}$$

where $\Phi = \Theta - \Omega t$.

This system will exhibit a *partially locked state*, studied in our previous work [5, 6]. For a non-zero coupling strength S, after a sufficient period of time at which the system reaches a stable state, there will exist two groups of oscillators with different behaviors. The oscillators in the *phase-locked group* will have the same frequency, and thus their phase differences will remain

constant at any given time point. The oscillators in the *drifting group* will not be able to *phase-lock* with the first group of oscillators and will have different frequencies. Thus, they will *drift* with different phase differences from a given time point to a next time point. The condition for the oscillators to be phase-locked is $\dot{\phi}_j = 0$. In order to satisfy this condition, the amplitude of the coupling terms must be larger than the inherent terms:

$$Sn_jR > |\omega_j - \Omega|. \tag{S7}$$

From the previous analysis of ref. [6], we know that for our set of parameters $\omega > 0$ and $\beta \in (0, \pi/2)$, $\omega_j - \Omega$ will be larger than zero. This means that the average frequency of the oscillators will be lower than the initially given frequencies: the oscillators slow down as they synchronize with each other. If we assume that the initial frequencies ω_j for each node j are given identically ($\omega_j = \omega$ for $j = 1, 2, ..., N$), we can write the following expression as the condition for the oscillator j to phase-lock:

$$n_j > \frac{\omega - \Omega}{SR} \equiv n_m. \tag{S8}$$

The oscillators that phase-lock are the ones with their $n_j > n_m$,

$$D_l = \left\{ j : n_m < n_j \right\}. \tag{S9}$$

The oscillators that drift are the ones with their $n_j < n_m$,

$$D_d = \left\{ j : n_j < n_m \right\}. \tag{S10}$$

The oscillators satisfying the condition (S9) will asymptotically approach a stable solution ${\phi_j}^*$ obtained from the following equation:

$$\omega - \Omega = Sn_jR \sin({\phi_j}^* - \Phi + \beta). \tag{S11}$$

We can rearrange this equation to the following form:

$$\phi_j^* = \sin^{-1}(\frac{\Delta}{Sn_jR}) + \Phi - \beta, \tag{S12}$$

where $\Delta = \omega - \Omega$.

Oscillators with the condition (S10) cannot satisfy the condition $\dot{\phi}_j = 0$. They will drift monotonically without locking. We can describe their behavior by invariant probability density $\rho(\phi, n)$ in the stationary state. In the stationary state the invariant probability density shall satisfy the condition $\rho(\phi, n)v = constant$, where v is the instantaneous frequency $\dot{\phi}$.

From this condition, we obtain the following probability density:

$$\rho(\phi, n) = \frac{C}{\Delta + SnR\sin(\Phi - \phi - \beta)}, \tag{S13}$$

where the normalization constant C can be calculated from $\int_0^{2\pi} \rho(\phi)d\phi = 1$:

$$C = \frac{1}{2\pi}\sqrt{\Delta^2 - (SnR)^2}. \tag{S14}$$

Equation (S12) gives the phase value for the phase-locked oscillators. Equation (S13) and (S14) give the phase distribution for the drifting oscillators. Both of the equations can be solved given R and Δ. R and Δ can be obtained in a similar way as described in ref. [5].

In the rotating frame of oscillator populations with the frequency Ω, the order parameter contribution from the locked subpopulation can be written as the following:

$$\begin{aligned} &\int_{D_l} dn\ g(n)e^{i\phi^*(n)} \\ &= e^{-i\beta}e^{i\Phi}\int_{D_l} dn\ g(n) \\ &\times \frac{\sqrt{(SnR)^2 - \Delta^2} + \Delta}{SnR}, \end{aligned} \tag{S15}$$

where $\mathcal{D}_l$ is the domain with $SnRN > |\Delta|$ and $g(n)$ is the distribution for n.

The order parameter contribution from the drifting subpopulation can be calculated by using population density $\rho(\phi, n)$ of Eq. (S13).

$$\begin{aligned}
&\int_{D_d}\int_0^{2\pi} d\phi\, dn\; g(n)\rho(\phi,n)e^{i\phi} \\
&= ie^{-i\beta}e^{i\Phi}\int_{D_d} dn\;\frac{g(n)}{SnR}\Big[\Delta \\
&\quad - \operatorname{sgn}(\Delta)\sqrt{\Delta^2-(SnR)^2}\,\Big],
\end{aligned} \tag{S16}$$

where $\int_0^{2\pi} d\phi\; \rho(\phi,n)e^{i\phi}$ is calculated using contour integration, and $\operatorname{sgn}(x)$ is the sign function. $\operatorname{sgn}(\Delta)$ appears for it determines which pole is inside the contour. $\mathcal{D}_d$ is the domain with $SnRN < |\Delta|$.

The sum of the contributions from the locked subpopulation (Eq. (S15)) and from the drifting subpopulation (Eq. (S16)) together constitutes the order parameter $Re^{i\Phi}$ in the rotating frame. For R and Φ independent of n, we obtain the following relation:

$$\begin{aligned}
R^2 \;=\; & ie^{-i\beta}\Big[\int_{D_{tot}}\frac{g(n)}{Sn}\Delta\, dn \\
& -i\int_{D_l}\frac{g(n)}{Sn}\sqrt{(SnR)^2-\Delta^2}\, dn \\
& -\int_{D_d}\frac{g(n)\operatorname{sgn}(\Delta)}{Sn}\sqrt{\Delta^2-(SnR)^2}\, dn\Big],
\end{aligned} \tag{S17}$$

where $\mathcal{D}_{tot}$ is the total range of n. This gives two independent equations for R and Δ, which can be solved numerically.

Local order parameter method applied to the model

In this section we introduce *local order parameter method* to calculate the phase for equation (S2). A similar method has been used for other systems [8], and/or other models [9].

We begin by introducing *local order paramter r*:

$$r_j e^{i\Theta_j} = \frac{1}{n_j}\sum_{k=1}^{N} A_{jk}e^{i\theta_k}. \tag{S18}$$

where n_j is again the sum of coupling to the oscillator j defined as $n_j = \sum_{k=1}^{N} A_{jk}$. Local order parameter r_j of the oscillator j is a measure of synchrony among the oscillators connected to the oscillator j. The value will be 1 if they are in perfect synchrony and 0 if they are incoherent. Using the local order parameter r_j, we can rewrite equation (S2) to the following form:

$$\dot{\theta}_j = \omega_j + S n_j r_j \sin(\Theta_j - \theta_j - \beta), \qquad j = 1, 2, ..., N. \tag{S19}$$

Unlike equation (S4) and (S6) using MFA, this equation (S19) is an exact restatement of the original equation (S2). The aim of this section is to solve this equation (S19) for the phase of each oscillator j, θ_j.

We can use the change of variables utilizing Ω (the frequency of the population oscillation of equation (S5) after the system approaches a stationary state). By letting $\phi_j = \theta_j - \Omega t$, and $\Phi_j = \Theta_j - \Omega t$, equation (S19) can be rewritten:

$$\dot{\phi}_j = \omega_j - \Omega + S n_j r_j \sin(\Phi_j - \phi_j - \beta), \qquad j = 1, 2, ..., N. \tag{S20}$$

Again, the condition for the oscillators to be phase-locked is $\dot{\phi}_j = 0$. The amplitude of the coupling terms for the oscillators must be larger than the inherent terms:

$$S n_j r_j > |\omega_j - \Omega|. \tag{S21}$$

For our set of parameters $\omega > 0$ and $\beta \in (0, \pi/2)$, $\omega_j - \Omega$ will be larger than zero [6]. If we assume that the initial frequencies ω_j for each node j are given identically ($\omega_j = \omega$ for $j = 1, 2, ..., N$), we can write the following expression as the condition for the oscillator j to phase-lock:

$$n_j r_j > \frac{\omega - \Omega}{S} \equiv n_l. \tag{S22}$$

The oscillators that phase-lock are the ones with their $n_j > n_l$,

$$D_l = \left\{ j : n_l < n_j r_j \right\}. \tag{S23}$$

The oscillators that drift are the ones with their $n_j < n_l$,

$$D_d = \left\{ j : n_j r_j < n_l \right\}. \tag{S24}$$

Unlike the condition for the case of the MFA, now the condition involves the local order parameter r_j. It is no longer the case that the degree (or the weighted degree in the case of the weighted network) of the oscillator j, n_j solely determines the dynamics of the oscillator j. Now the connected oscillators play a role via r_j. Therefore, even though an oscillator has a degree less than n_m (the threshold for the MFA), it is now possible to phase-lock if the oscillator has a large value of r_j.

From equation (S20), we can derive the exact expression for the phase of oscillator j. The oscillators satisfying the condition (S23) will asymptotically approach a stable solution ${\phi_j}^*$ obtained from the following equation:

$$\omega - \Omega = S n_j r_j \sin({\phi_j}^* - \Phi_j + \beta). \tag{S25}$$

Rearranging this equation, we arrive at the following form:

$$\phi_j^* = \sin^{-1}\left(\frac{\Delta}{S n_j r_j}\right) + \Phi_j - \beta, \tag{S26}$$

where $\Delta = \omega - \Omega$.

The oscillators with the condition (S24) cannot satisfy the condition $\dot{\phi}_j = 0$ and drift monotonically without locking. Their behavior is described by the invariant probability density ρ and the condition $\rho v = constant$ which should be satisfied in the stationary state. v is the instantaneous frequency $\dot{\phi}$.

The probability density is obtained from the following condition:

$$\rho(\phi) = \frac{C}{\Delta + S n_j r_j \sin(\Phi_j - \phi - \beta)}, \tag{S27}$$

and the normalization constant C can be calculated from $\int_0^{2\pi} \rho(\phi) d\phi = 1$:

$$C = \frac{1}{2\pi}\sqrt{\Delta^2 - (S n_j r_j)^2}. \tag{S28}$$

Equation (S26) gives the exact phase value for the phase-locked oscillators. Equation (S27) and (S28) give the phase distribution for the drifting oscillators. These equations can now be solved in a similar way to that described in ref. [10, 11].

In the rotating frame of the oscillator populations with the frequency Ω, the local order parameter contribution from a locked subpopulation is written as the following:

$$\begin{aligned}&\frac{1}{n_j}\sum_{k\in D_l} A_{jk}e^{i\phi_k^*}\\&=\frac{e^{-i\beta}}{n_j}\sum_{k\in D_l} A_{jk}e^{i\Phi_k}\times\frac{\sqrt{(Sn_kr_k)^2-\Delta^2}+i\Delta}{Sn_kr_k}.\end{aligned}\tag{S29}$$

The local order parameter contribution from the drifting subpopulation can be calculated by using population density $\rho(\phi)$ of Eq. (S27).

$$\begin{aligned}&\frac{1}{n_j}\sum_{k\in D_d} A_{jk}\int_0^{2\pi} d\phi\ \rho(\phi)e^{i\phi}\\&=\frac{ie^{-i\beta}}{n_j}\sum_{k\in D_d} A_{jk}\frac{e^{i\Phi_k}}{Sn_kr_k}\times\left[\Delta-\sqrt{\Delta^2-(Sn_kr_k)^2}\ \right]\end{aligned}\tag{S30}$$

where $\int_0^{2\pi} d\phi\ \rho(\phi)e^{i\phi}$ is calculated using contour integration.

The sum of the contributions from the locked subpopulation (Eq. (S29)) and from the drifting subpopulation (Eq. (S30)) constitutes the local order parameter $r_je^{i\Phi_j}$ in the rotating frame. For r_j, Φ_j and Δ, we obtain the following relation:

$$\begin{aligned}r_je^{i\Phi_j} &= \frac{ie^{-i\beta}}{n_j}\left[\sum_{k=1}^{N} A_{jk}\frac{e^{i\Phi_k}\Delta}{Sn_kr_k} - i\sum_{k\in D_l} A_{jk}\frac{e^{i\Phi_k}\sqrt{(Sn_kr_k)^2-\Delta^2}}{Sn_kr_k}\right.\\&\qquad\left.-\sum_{k\in D_d} A_{jk}\frac{e^{i\Phi_k}\sqrt{\Delta^2-(Sn_kr_k)^2}}{Sn_kr_k}\right].\end{aligned}\tag{S31}$$

This equation can be solved numerically, giving values to the unknowns r_j, Φ_j and Δ [10, 11].

Relationship between directed Phase Lag Index and phase

We used directed phase lag index (dPLI) as the measure of *directionality* between oscillators [12]. This measure reflects which of the two signals is leading and which is lagging in phase. dPLI is defined as:

$$dPLI_{jk} = \langle sign\{\Delta\theta_{jk}(t)\}\rangle_t, \tag{S32}$$

Here, $sign\{\}$ function is defined to give either 1 if the argument of the function is positive, -1 if the argument is negative, and 0 if the argument is 0. $\Delta\theta_{ij}(t)$ is the instantaneous phase difference between two nodes i and j: $\Delta\theta_{ij}(t) = \theta_i(t) - \theta_j(t)$. Thus $sign\{\}$ function will yield 1 if $\Delta\theta_{ij}(t) > 0$, -1 $\Delta\theta_{ij}(t) < 0$, and 0 if $\Delta\theta_{ij}(t) = 0$. The mean $\langle\rangle_t$ is taken over all t=1, 2, 3, ..., n. Therefore, if on average, node i leads node j, $0 < dPLI_{ij}1$; if node j leads node i, $-1dPLI_{ij} < 0$; and if there is no phase-lead/phase-lag relationship between nodes, $dPLI_{ij} = 0$.

Given two oscillators, if they are phase-locked with each other, their phase difference can be written as the following:

$$|\Delta\theta_{jk}| = |\theta_j - \theta_k| = constant, \tag{S33}$$

for any given time point $t = 1, 2, ..., T$. Therefore, given two phase-locked oscillators,

$$\begin{aligned} &if \quad \theta_j \quad \textit{phase-leads} \quad \theta_k, \quad then \quad dPLI_{jk} = 1, \\ &if \quad \theta_j \quad \textit{phase-lags} \quad \theta_k, \quad then \quad dPLI_{jk} = -1. \end{aligned} \tag{S34}$$

For each node θ_j, $dPLI_j$ is defined as the average of $dPLI_{jk}$ for all other nodes k:

$$dPLI_j = \frac{1}{N}\sum_{k=1}^{N} dPLI_{jk} = \frac{1}{N}\sum_{k=1}^{N}\langle sign\{\Delta\theta_{jk}(t)\}\rangle_t. \tag{S35}$$

For each node j, the transformed phase $\phi_j = \theta_j - \Omega t$ represents the phase relative to the average oscillation:

$$\phi_j = \theta_j - \Omega t = \theta_j - \frac{1}{N}\sum_{k=1}^{N}\theta_k = \frac{1}{N}\sum_{k=1}^{N}\Delta\theta_{jk}. \tag{S36}$$

For phase-locked oscillators, the phase differences between oscillators do not change over time. Therefore, the time-averaged value of $dPLI_{jk}$ will be equal to the value of $dPLI_{jk}$ at any given time point:

$$dPLI_j = \frac{1}{N}\sum_{k=1}^{N} sign\{\Delta\theta_{jk}\}. \tag{S37}$$

For phase-locked oscillators, the difference between $dPLI_j$ and ϕ_j is reduced to the $sign\{\}$ function. We can think of $dPLI_j$ as a counting function of how often ϕ_j phase-leads or phase-lags other oscillators, whereas ϕ_j is a *weighted* counting function taking into account how much they are leading or lagging. Therefore, for most probability distributions of the phase differences $\Delta\theta_{jk}$, these two measures will yield qualitatively similar values for each oscillator j. Only a very skewed probability distribution functions will yield qualitatively different values.

2 Experimental analysis

In this section, we provide more information on the experimental analysis of brain networks of different species. First we describe in depth the process of constructing the structural brain network of mouse.

Construction of the mouse structural network

Image acquisition
In vivo DTI of adult mouse brains (n = 8) was performed using a modified 3D diffusion- weighted gradient and spin echo (DW-GRASE) sequence [13] with the following parameters: TE/TR = 33/500 ms, 2 signal averages, 20 imaging echoes (4 spin echoes distributed along the phase encoding direction and 16 gradient echoes distributed along the slice selection direction) after each excitation with twin navigator echoes in the end for motion and phase corrections, 12 diffusion directions, b = 1000 s/mm2, field of view (FOV) = 16 mm × 16 mm × 17.6 mm, a matrix size of 128 × 128 × 140, and a native imaging resolution = 0.125 mm × 0.125 mm × 0.125 mm. This dataset is available at the Johns Hopkins Medical Institute, Laboratory of Brain Anatomical MRI, and was downloaded from http://cmrm.med.jhmi.edu [15]. All experimental

procedures were approved by the Animal Use and Care Committee at the Johns Hopkins University School of Medicine.

Image Processing

The 3D images acquired using the DW-GRASE sequence were reconstructed from raw data in MATLAB (www.mathworks.com) with navigator-based motion and phase correction [14]. Using the log-linear fitting method implemented in DTIStudio (http://www.mristudio.org) [16], diffusion tensor was calculated at each pixel along with the apparent diffusion coefficient (ADC), fractional anisotropy (FA), primary eigenvector, axial diffusivity (‖, the primary eigenvalue), and radial diffusivity (, the average of the secondary and tertiary eigenvalues). The adult mouse brain images were rigidly aligned to ex vivo mouse brain images in our MRI based mouse brain atlas [17] using the landmark based rigid transformation implemented in the DiffeoMap software (www.mristudio.org). In addition, we took the image volumes representing the canonical Waxholm Space (WHS) mouse brain [18], which include T1-, T2*-, and T2-Weighted MR volumes, Nissl-stained optical histology, and a label volume describing 37 structures (all volumes are represented at 21.5m isotropic resolution and are available at http://software.incf.org/software/waxholm-space). From the defined 37 structures we selected 24 gray matter regions. We separated left and right hemispheres, and because in this parcellation scheme the cerebral cortex is originally denoted as only one region, we reparcellated both hemispheric cerebral cortex into 50 small regions (i.e., 50 for each hemispheric cortex) of approximately the same volume [19]. Finally, the parcellation procedure resulted in a modified WHS parcellation scheme of 74 cortical and subcortical gray matter regions for each hemisphere.

Axonal connectivity estimation and Network Construction

Axonal trajectories between each pair of gray matter regions (defined by the modified WHS parcellation scheme) were estimated using a fully automated fiber tractography [20]. Tracking parameters used were: 200 mm as maximum trace length and 900 as curvature threshold over voxel. Next, a whole brain undirected weighted network was created as follows: 1) a node was defined to represent each considered anatomic region, 2) an undirected arc aij between any nodes i and j was established with a corresponding arc weight w(aij), defined as the effective number of connected voxels (weighing by the obtained probability of connection) relative to the number of voxels over the surface of regions i and j, where each fiber path was quantified according to

the arithmetic mean of the inverse of its mean diffusivity values. Finally, the connectivity backbone was estimated for the created brain network [19, 21]. First, a maximum spanning tree, which connects all nodes of the network such that the sum of its weights is maximal, was extracted; then, additional edges were added in order of their weight until the average node degree was 4. All posterior network analysis and visual representations were based on the resultant networks (connectivity backbones).

3 Supplementary Figures Information

Supplementary Figure 1
In our work, the analytically calculated phases and dPLIs were directly compared with those of EEG data from humans, macaques, and mice. For the visualization, we first mapped the averaged node degree, phase and dPLI values at each node to their positions in the brains, both for analytical predictions and experimental analysis. Then the mapped values were extrapolated to make the topographic figures in Figure 4 of the main manuscript for all subjects. The pre-extrapolated channel level data are shown in Supplementary Figure 1, for both analytical prediction (left column) and experimental analysis (right column).

Supplementary Figure 2
For macaque monkeys (three M. fuscata, and one M. mulatta), the data were freely acquired from Project Tycho (http://neurotycho.org/) from RIKEN [22]. For each monkey, 128-channel electrocorticography (ECoG) of a single (left) hemisphere was recorded in the eyes-closed resting state. Since the four monkeys all have slightly different channel locations, we divided the left hemisphere of the cortex into 48 regions by using the parcellation scheme developed by Lewis and van Essen, and calculated the average value of channels for each region [23]. The 48 regional values were then compared across monkeys. The parcellation scheme is shown is Supplementary Figure 2. The dotted lines in gray represent the borders between each region. As an example, the dPLI values at each channel for all monkeys are shown in the figure, before the averaging process.

Supplementary Figure 3
We compared the values from the analytic prediction from mean-field approx-

imation (MFA) method, local order parameter (LOP) method, and experimental analysis (EXP). We computed Spearman correlations for the degree, phase and dPLI values between each analysis. The results are shown in Supplementary Figure 3. Each element in the matrices represents the correlation values between each analysis. Note that the highest correlation value between different analysis was LOP and EXP for the dPLI of human left hemisphere (0.89). The lowest value was between LOP and EXP for the phase of macaque (0.28).

Supplementary Figure 4-7
The topography figures for the data of individual subjects from human left hemisphere and entire hemisphere (Supplementary Figure 4 and 5), macaque (Supplementary Figure 6) and mouse (Figure 7) are shown, before the averaging was performed (Figure 4 in the main manuscript shows the results after averaging across subjects). We note that a few individual subjects show opposite patterns such as the phase and dPLI of human subjects 2 and 4, and the degree of mouse subject 2. These deviations are expected, since the predictions are all done based on structural networks averaged over a large number of subjects for each species, and the experimental data are from different subjects altogether. Therefore our prediction would become more accurate when compared to empirical data averaged over a large number of subjects. Simultaneous recordings of both anatomical network and EEG for the same individual could also improve the predictability.

Supplementary Figure 8
We also performed our simulation with distance-dependent delays between each node of the brain network. The topography figures are shown for phases and dPLIs in Supplementary Figure 8 (a) and (b). We also compared the values from the analytic prediction from local order parameter (LOP) method, distance-dependent delays simulations (SIM), and experimental analysis (EXP). We computed Spearman correlations for the phase and dPLI values between each analysis. The results are shown in Supplementary Figure 8 (c) and (d). Each element in the matrices represents the correlation values between each analysis. Note that the correlation values between SIM and EXP are usually higher than the values between LOP and EXP.

Supporting References

[1] Hoppensteadt, F. C. & Izhikevich, E. M. *Weakly Connected Neural Networks.* (Springer-Verlag, 1997).

[2] Kuramoto, Y. *International Symposium on Mathematical Problems in Theoretical Physics, Lecture Notes in Physics, 39.* (Arakai, H.), 420-422 (Springer-Verlag, 1975).

[3] Strogatz, S. H. *Nonlinear Dynamics and Chaos. 1st ed.* (Westview Press, 1994).

[4] Izhikevich, E. M. Phase models with explicit time delays. *Phys Rev E.* **58**, 905-908 (1998).

[5] Ko, T. W. & Ermentrout, G. B. Partially locked states in coupled oscillators due to inhomogeneous coupling. *Phys Rev E.* **78**, 016203 (2008).

[6] Moon, J.-Y., Lee, U., Blain-Moraes & S., Mashour, G. A. General relationship of global topology, local dynamics, and directionality in large-scale brain networks. *PLoS Comput Biol.* **11**, e1004225 (2015).

[7] Hong, H., Park, H. & Tang, L.-H. Finite-size scaling of synchronized oscillation on complex networks. *Phys Rev E.* **76**, 066104 (2007).

[8] Shima, S. I. & Kuramoto, Y. Rotating spiral waves with phase-randomized core in nonlocally coupled oscillators. *Phys. Rev. E.* **69**, 036213 (2004).

[9] Restrepo, J. G., Ott, E. & Hunt, B. R. Onset of synchronization in large networks of coupled oscillators. *Phys. Rev. E.* **71**, 036152 (2004).

[10] Kuramoto, Y. & Battogtokh, D. Coexistence of coherence and incoherence in nonlocally coupled phase oscillators. *Nonlin. Phenom. Compl. Syst.* **5**, 380-385 (2002).

[11] Abrams, D. M. & Strogatz, S. H. Chimera states in a ring of nonlocally coupled oscillators. *Int. J. Bifurc. Chaos.* **16**, 21–37 (2006).

[12] Stam, C. J. & van Straaten, E.C. W. Go with the flow: use of a directed phase lag index (dPLI) to characterize patterns of phase relations in a large-scale model of brain dynamics. *NeuroImage.* **62**, 1415-1428 (2012).

[13] Aggarwal, M., Mori, S., Shimogori, T., Blackshaw, S. & Zhang J. Three-dimensional diffusion tensor microimaging for anatomical characterization of the mouse brain. *Magn Reson Med.* **64**, 249–261 (2010).

[14] Mori, S. & van Zijl, P. C. A motion correction scheme by twin-echo navigation for diffusion-weighted magnetic resonance imaging with multiple RF echo acquisition. *Magn Reson Med.* **40**, 511-516 (1998).

[15] Wu, D., Xu, J., McMahon, M. T., van Zijl, P. C., Mori, S., Northington, F. J. & Zhang, J. In vivo high-resolution diffusion tensor imaging of the mouse brain. *Neuroimage.* **83**, 18-26 (2013).

[16] Jiang, H., van Zijl, P. C., Kim, J., Pearlson, G. D. & Mori, S. DtiStudio: resource program for diffusion tensor computation and fiber bundle tracking. *Comput Methods Programs Biomed.* **81**, 106–116 (2006).

[17] Chuang, N., Mori, S., Yamamoto, A., Jiang, H., Ye, X., Xu, X., Richards, L. J., Nathans, J., Miller, M. I., Toga, A. W., Sidman, R. L. & Zhang, J. An MRI-based atlas and database of the developing mouse brain. *NeuroImage.* **54**, 80–89 (2011).

[18] Johnson, G., Badea, A., Brandenburg, J., Cofer, G., Fubara, B. *et al.* Waxholm space: an image-based reference for coordinating mouse brain research. *Neuroimage.* **53**, 365-72 (2010).

[19] Iturria-Medina, Y., Pérez Fernández, A., Valdés Hernández, P., García Pentón, L., Canales-Rodríguez, E. J., Melie-Garcia, L., Lage Castellanos, A. & Ontivero Ortega, M. Automated discrimination of brain pathological state attending to complex structural brain network properties: the shiverer mutant mouse case. *PLoS One.* **6**, e19071 (2011).

[20] Iturria-Medina, Y., Canales-Rodríguez, E. J., Melie-García, L., Valdés-Hernández, P. A., Martínez-Montes, E., Alemán-Gómez, Y. & Sánchez-Bornot, J. M. Characterizing brain anatomical connections using diffusion weighted MRI and graph theory. *Neuroimage.* **36**, 645-60 (2007).

[21] Hagmann, P., Cammoun, L., Gigandet, X., Meuli, R., Honey, C. J. *et al.* Mapping the structural core of human cerebral cortex. *Plos Biology.* **6**, 1479–1493 (2008).

[22] Yanagawa, T., Chao, Z. C., Hasegawa, N. & Fujii, N. Large-scale information flow in conscious and unconscious states: an ECoG study in monkeys. *PLoS One.* **8**, e80845 (2013).

[23] Lewis, J. W. & Van Essen, D. C. Corticocortical connections of visual, sensorimotor, and multimodal processing areas in the parietal lobe of the macaque monkey. *J. Comp. Neurol.* **428**, 112–37 (2000).

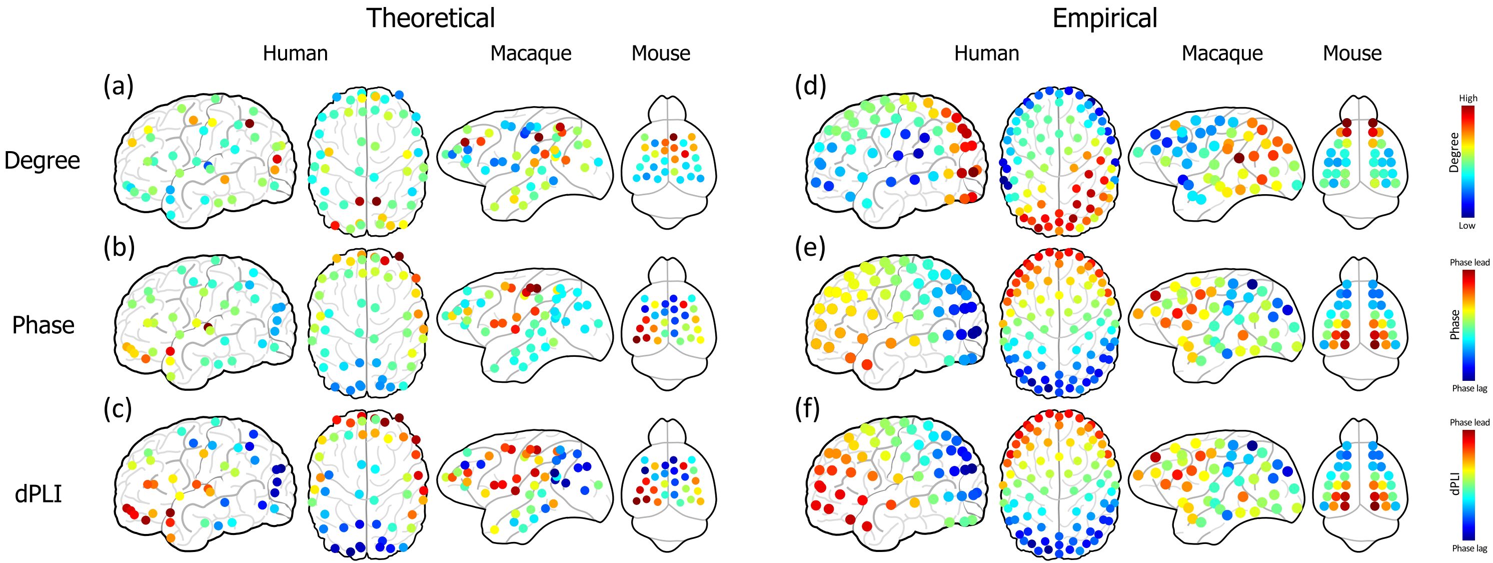

Supplementary Figure 1: Channel plots of degree, phase, and directed Phase Lag Index (dPLI)for brain networks. Channel plot figures from theoretical prediction(a)-(c), and empirical analysis (d)-(f) are shown for the brain networks of each species. Degree calculated from (a) structural network and (d) functional network from PLI analysis of EEG/ECoG for different species are shown. Phase predicted from (b) theory and (e) experiments of different species are shown.dPLI predicted from (c) theory, and (f) experiments for different species are shown.

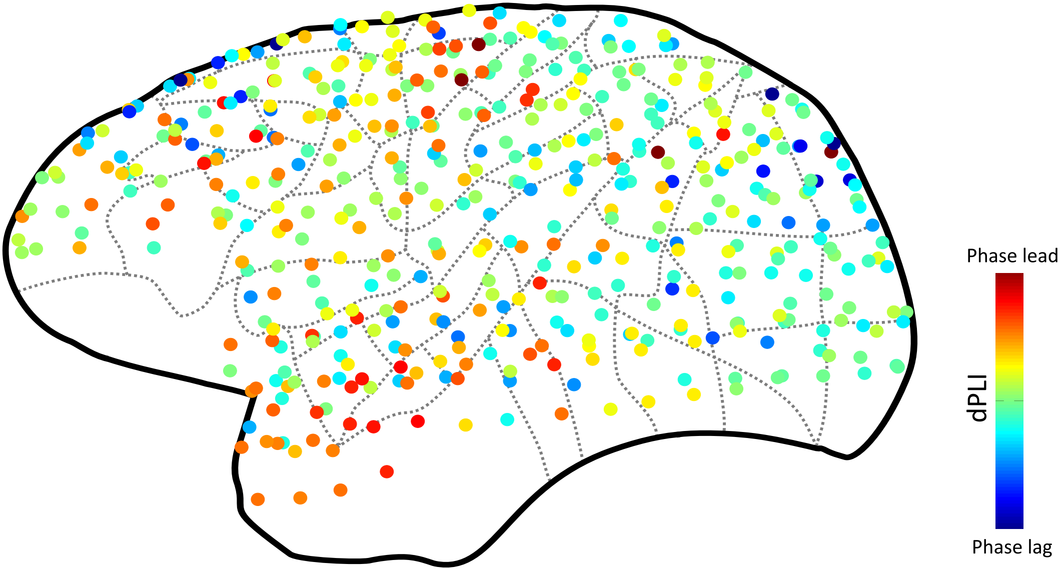

Supplementary Figure 2: Parcellation of the macaque brain. The parcellation scheme for the macaque brain by Lewis and Essen from ref. 18 is shown as gray dotted lines (---). The dPLI values for each channel for all 4 macaque subjects are shown here with the colors ranging from blue (small dPLI value) to red (large dPLI value). The channel locations are different from subject to subject.

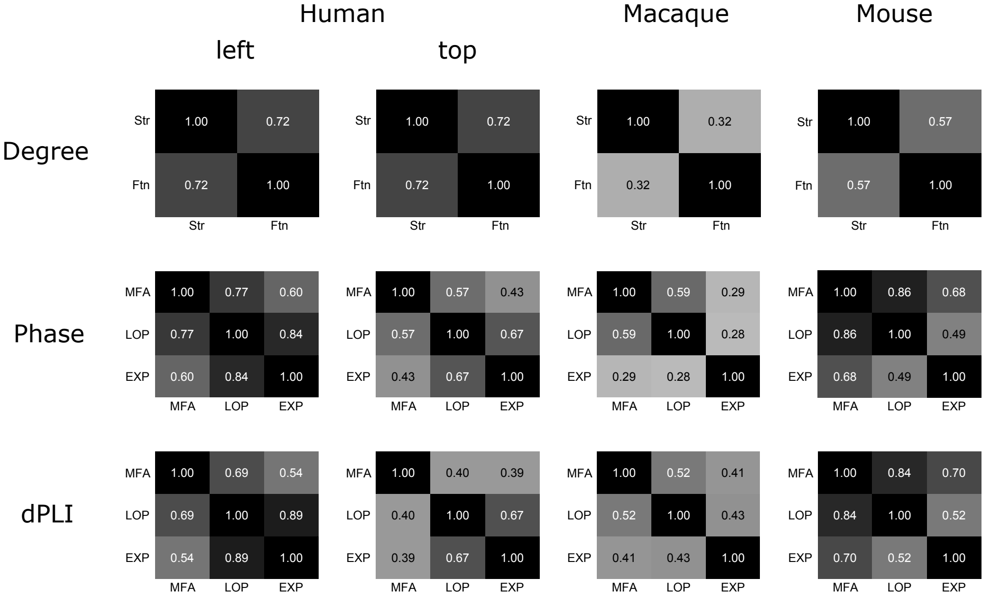

Supplementary Figure 3: Spearman correlation values between analytical prediction and experimental data. Each matrix elements shows the corresponding Spearman correlation between each dataset. The higher the value is for each element, the darker the color for the background of the element. The following abbreviations are used throughout: Str (structural), Ftn (functional), EXP (experimental), MFA (mean-field approximation), and LOP (local order parameter).

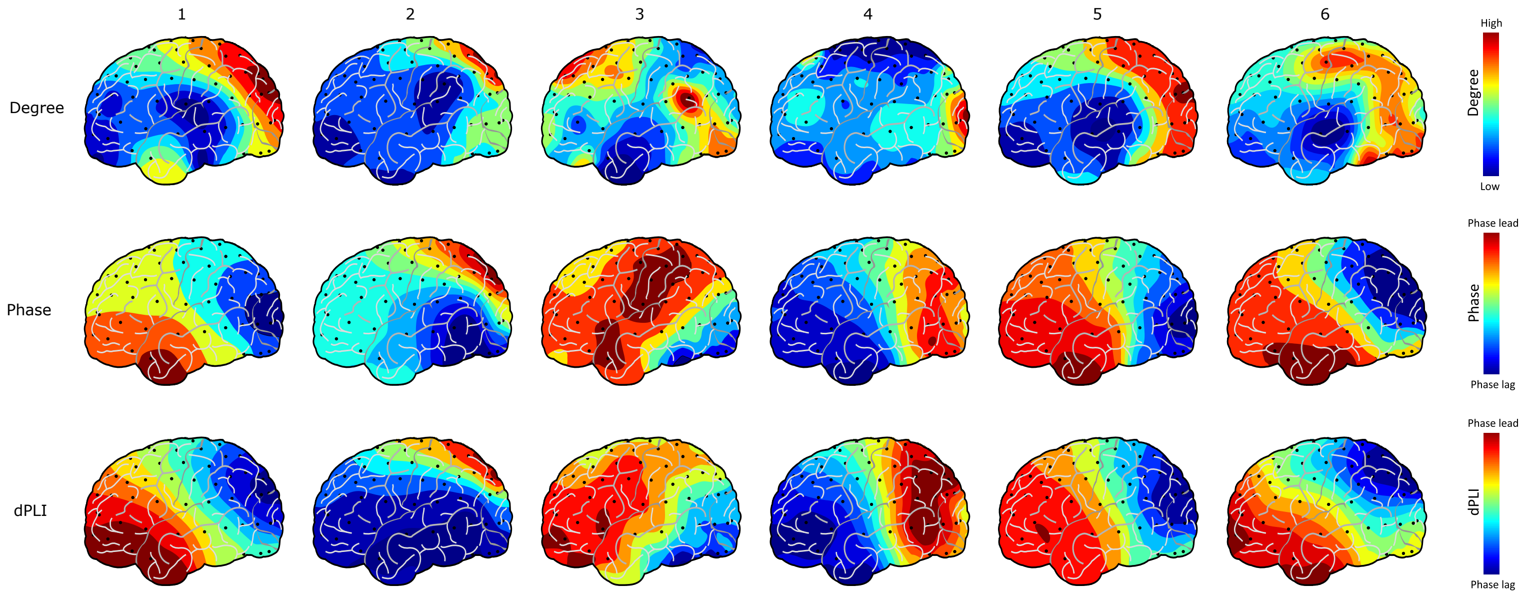

Supplementary Figure 4: Topographic plots of degree, phase, and dPLIfor brain networks of individual human subjects – side view. Topographic figures from empirical analysis of individual human subjects are shown, from left side view.

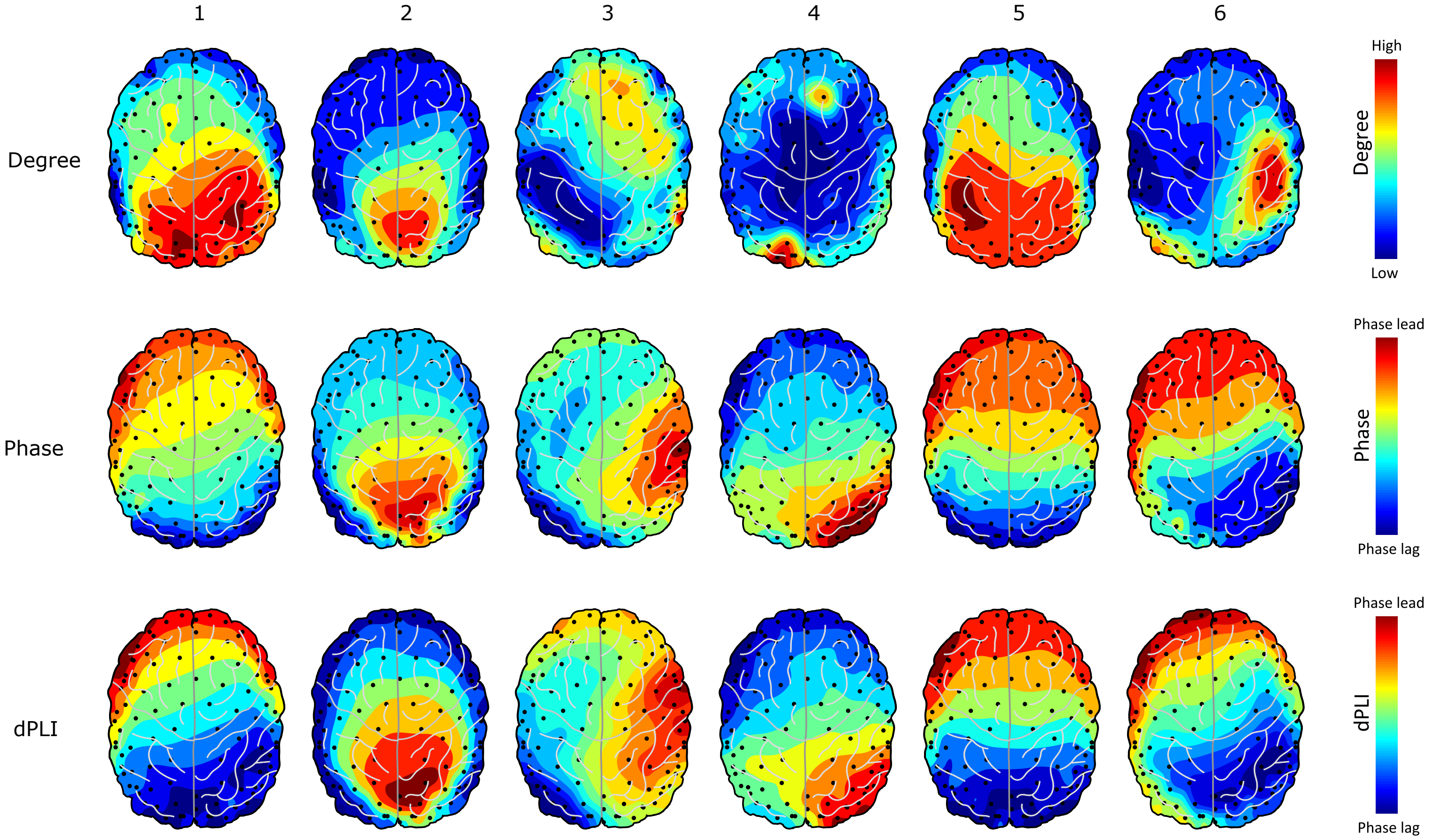

Supplementary Figure 5: Topographic plots of degree, phase, and dPLIfor brain networks of individual human subjects – top view. Topographic figures from empirical analysis of individual human subjects are shown, from top view.

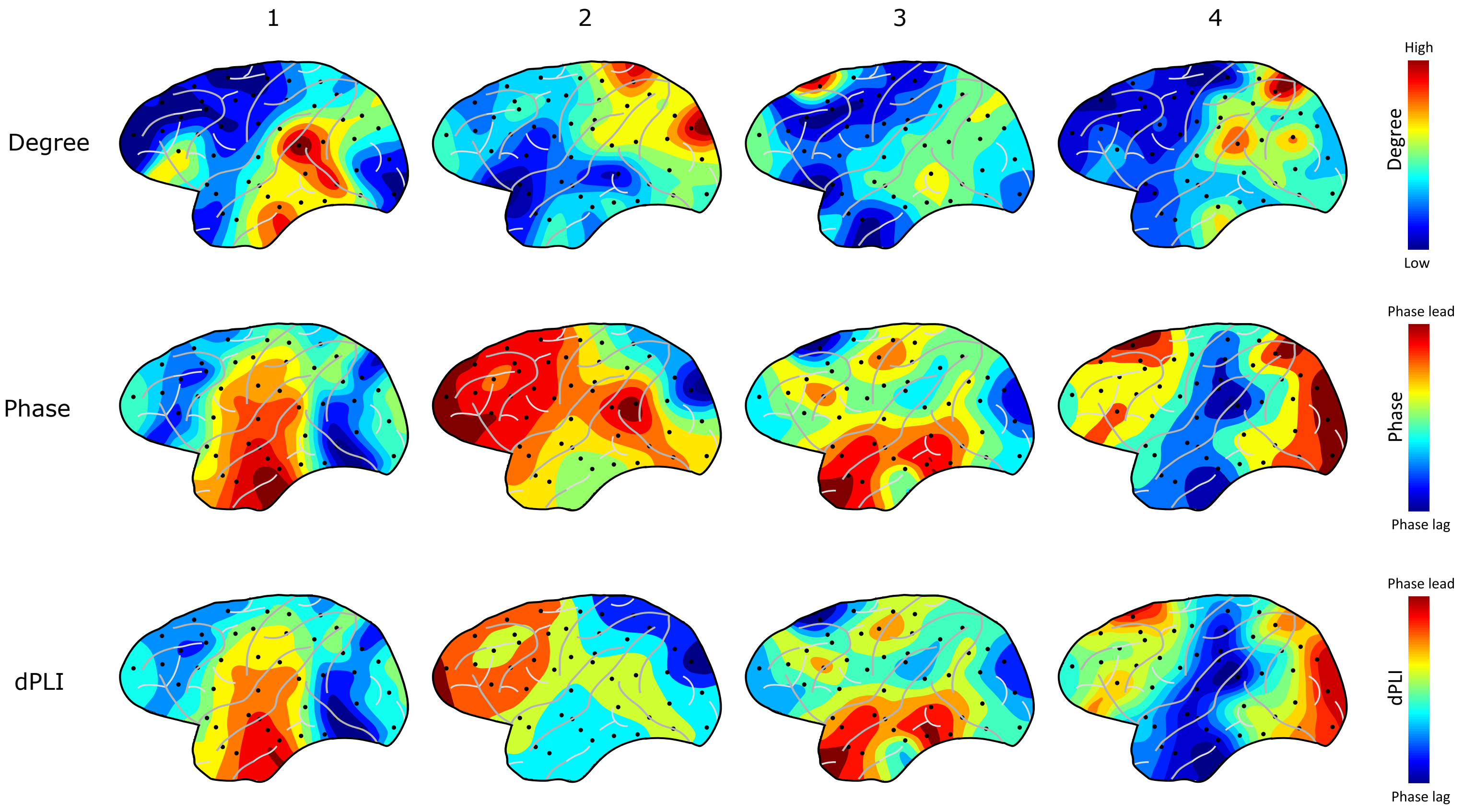

Supplementary Figure 6: Topographic plots of degree, phase, and dPLI for brain networks of individual macaque. Topographic figures from empirical analysis of individual macaque subjects are shown, from left side view.

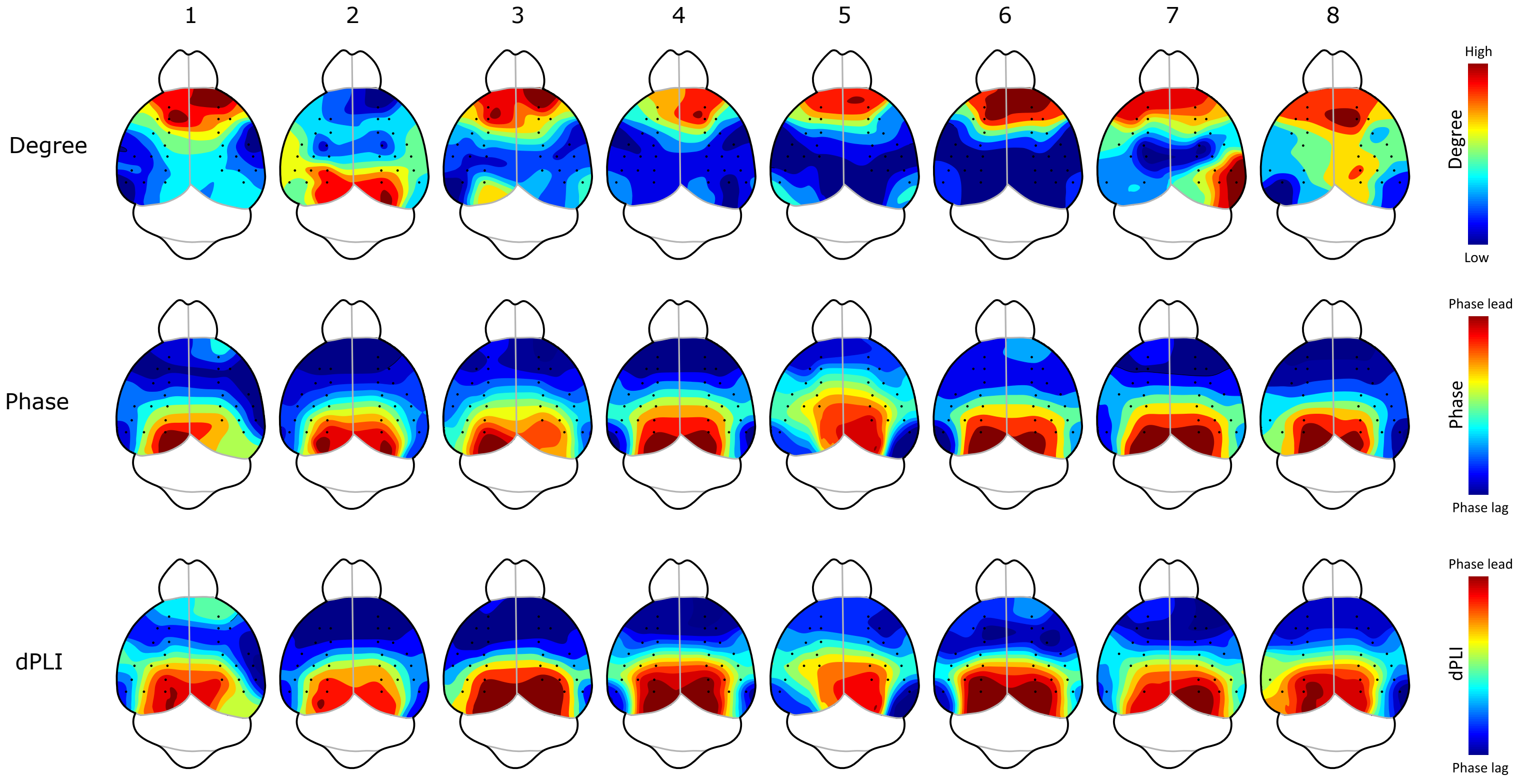

Supplementary Figure 7: Topographic plots of degree, phase, and dPLI for brain networks of individual mouse. Topographic figures from empirical analysis of individual mouse subjects are shown, from top view.

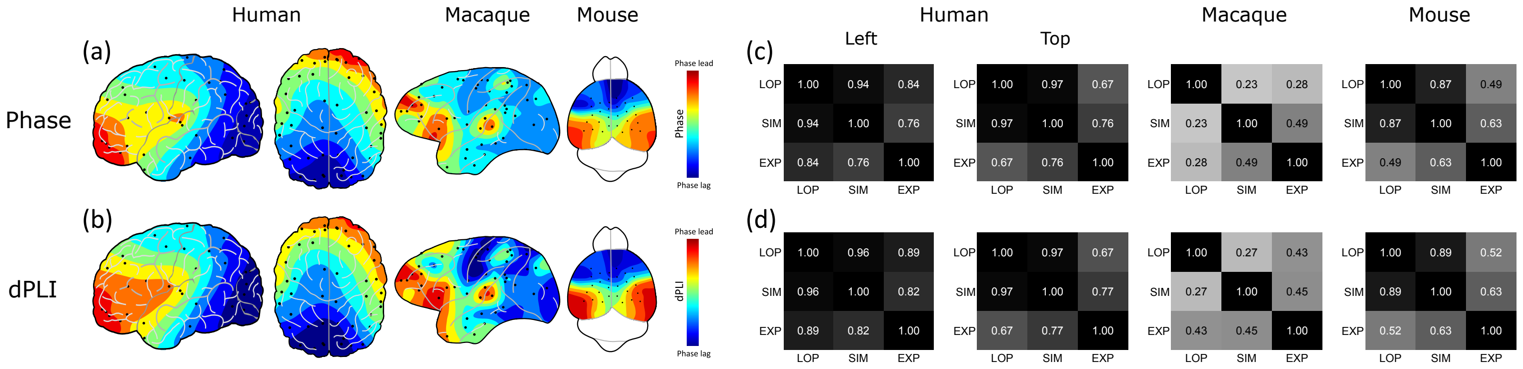

Supplementary Figure 8: Topographic plots of phase and dPLI for the heterogeneous time delay simulations as well as Spearman correlation values between the simulations and experimental data. (a) and (b) are the topographic plots for node phase and dPLI for the distance-dependent time delay simulations of human, macaque, and mouse. (c) and (d) are the Spearman correlation values between the analytic predictions using the local order parameter method (LOP), the distance-dependent time delay simulations (SIM) and the experimental data (EXP). Each matrix element shows the corresponding Spearman correlation between each dataset. The higher the value is for each element, the darker the color for the background of the element.
